# Shepherding in a Self-gravitating Disk of Trans-Neptunian Objects

**DOI:** 10.3847/1538-3881/aaf0fc

**Published:** 2019-01-21

**Authors:** Antranik A. Sefilian, Jihad R. Touma

**Affiliations:** 1 Department of Applied Mathematics and Theoretical Physics, University of Cambridge, Centre for Mathematical Sciences, Wilberforce Road, Cambridge CB3 0WA, UK; aas79@damtp.cam.ac.uk; 2 Department of Physics, American University of Beirut, PO BOX 11-0236, Riad El-Solh, Beirut 11097 2020, Lebanon; jt00@aub.edu.lb

**Keywords:** celestial mechanics, Kuiper belt: general, planets and satellites: dynamical evolution and stability

## Abstract

A relatively massive and moderately eccentric disk of trans-Neptunian objects (TNOs) can effectively counteract apse precession induced by the outer planets, and in the process shepherd highly eccentric members of its population into nearly stationary configurations that are antialigned with the disk itself. We were sufficiently intrigued by this remarkable feature to embark on an extensive exploration of the full spatial dynamics sustained by the combined action of giant planets and a massive trans-Neptunian debris disk. In the process, we identified ranges of disk mass, eccentricity, and precession rate that allow apse-clustered populations that faithfully reproduce key orbital properties of the much-discussed TNO population. The shepherding disk hypothesis is, to be sure, complementary to any potential ninth member of the solar system pantheon, and could obviate the need for it altogether. We discuss its essential ingredients in the context of solar system formation and evolution, and argue for their naturalness in view of the growing body of observational and theoretical knowledge about self-gravitating disks around massive bodies, extra-solar debris disks included.

## 1. Introduction

Trans-Neptunian (phase)-space appears to be populated with bodies that show signs of orbital sculpting, then shepherding. With the discovery of 2012 VP_113_, a Sedna-like object, Trujillo & Sheppard (2014) first argued for a ninth planet of 5 *M*_⊕_ on a circular orbit at 200 au as a potential shepherd of several trans- Neptunian objects (TNOs) with eccentric and inclined orbits showing peculiar clustering in the argument of periapse. Later on, Batygin & Brown (2016) noted remarkable spatial nodal alignment of the same objects. They reexamined the proposition of an additional planet, and argued instead for a super-Earth (dubbed “Planet Nine”) on a larger eccentric and inclined orbit, while appealing to an alternative resonant process for the aligning trap (Batygin & Brown 2016). Further indirect evidence for such a planet was sought around apparent deviations in the orbit of the Cassini spacecraft (Fienga et al. 2016), and in the potential to explain the Sun’s obliquity (Bailey et al. 2016; Lai 2016).

To date (i.e., submission date), 23 TNOs have been identified on eccentric and inclined orbits, with semimajor axes *a*_p_ $\underset{\sim }{≻}$ 150 au and perihelion distance *q_p_* > 30 au. Out of these, 13 roam with *a* $\underset{\sim }{≻}$ 250 au and have had their notorious kinematic properties classified in the course of Planet Nine- related studies, which propose to explain them. They are interpreted as either: spatially clustered and antialigned with Planet Nine (10 objects); spatially clustered and aligned with Planet Nine (two objects); neither here nor there, though strongly perturbed by Planet Nine (one object). These classes are, of course, expected to grow in size and definition by proponents of a ninth planet, which is required to structure, along with the rest of the solar system, the phase space in which TNOs are presumed to evolve.

Alternatively, Shankman et al. (2017a) argued that the spatial clustering that Planet Nine is supposed to explain is fraught with observational bias. Running their own orbital simulations, they disputed the claim that a planet alone could maintain clustering for the required duration. They further noted that observing this group of TNOs within existing campaigns implies a parent population of 6–24 *M*_⊕_. Such a massive reservoir of trans-Neptunian icy bodies is nearly two orders of magnitude larger than currently favored estimates (Gladman et al. 2011). Shankman et al. (2017a) took this requirement as further evidence against significant clustering, and gave no further consideration to the dynamical signature of a massive trans-Neptunian population.

Here, we go precisely after the dynamical impact of an extended and relatively massive disk of TNOs, and demon- strate that this alone can provide a fair amount of shepherding, perhaps obviating the need for an extra planetary member in the solar system pantheon, but surely complementing it.

We describe our results in a progression of complexity around a fiducial razor-thin disk. We then comment briefly on parametric variations on such a disk and discuss its properties and their origin, together with the potential interplay between the dynamical features it stimulates, together with those associated with a hypothetical planet of a few Earth masses in the post-Neptunian realm.

## 2. Coplanar Dynamics

We study the secular, orbit-averaged coplanar dynamics of trans-Neptunian test particles characterized by their semimajor axis *a_p_*, eccentricity *e_p_*, and apsidal angle *ϖ_p_*, in the combined gravitational potential of: (a) the outer planets; and (b) a hypothetical extended disk, lying in the plane of the giant planets, built out of confocal eccentric apse-aligned orbits.

The outer planets are included via the quadrupolar potential of a sequence of fixed concentric circular rings. The coplanar disk is parameterized by its non-axisymmetric surface density Σ (Equation (17)), eccentricity profile *e_d_*, global apsidal angle *ϖ_d_* (fixed at π, except otherwise stated), and inner and outer boundaries *a_in_* and *a_out_*, respectively.

We work with disks that have power-law density/eccentricity profiles

(1) $$\sum_{d}\left(a_{d}\right)=\sum_{0}{\left(\frac{a_{\mathrm{out}}}{a_{d}}\right)}^{p}$$

and

(2) $$e_{d}\left(a_{d}\right)=e_{0}{\left(\frac{a_{\mathrm{out}}}{a_{d}}\right)}^{p}$$

for *a*_in_ ≤ *a*_d_ ≤ *a*_out_. Here, Σ_0_ and e_0_ are the pericentric surface density and eccentricity at the outer edge of the disk respectively. Surface density profiles with *p* < 2 (*p* > 2) are associated with disks that have more mass concentrated in the outer (inner) parts of the disk than in the inner (outer) regions. Total disk mass M_d_ can be estimated with $M_{d}\;≃\;2\pi \int_{a_{in}}^{a_{out}}\;\sum_{d}\left(a_{d}\right)a_{d}da_{d}$, yielding

(3) $$M_{d}=\frac{2\pi }{2-p}\Sigma_{0}a_{out}^{2}\left[1-{\left(\frac{a_{in}}{a_{out}}\right)}^{2-p}\right]\approx \frac{2\pi }{2-p}\Sigma_{0}a_{out}^{2}$$

where the approximation is valid as long as the disk edges are well-separated and more mass is found in the outer parts. Disk models that are thoroughly explored in this work are listed in Table 1.

**Table 1:** Power-law Disk Models

Disk Model	*P*	*q*	*a_in_* (au)	*a_out_* (au)	ϖ_d_(rd)	*e_0_*	*M_d_* (*M*_Å_)
DM1	0.5	-1	40	750	π	0.165	10
DM2	0.5	-1	40	750	π	0.165	2.5
DM3	0.5	-1	40	750	π	0.165	20
DM4	2.5	-1	40	750	π	0.165	10

**Note.** Disk Model 1 (DM1) is the fiducial disk configuration adopted in this work.

Our basic shepherding mechanism is articulated best in planar dynamics, which will ultimately provide the skeleton around which fully inclined behavior is structured (see Section 3).

At the outset, it is important to remind the reader that hot, nearly Keplerian disks induce negative apse precession in their constitutive particles, in contrast to the familiar prograde apse precession expected from cold disks of isolated planets. This fact was recently cited to argue for the role of massive gaseous disks in mitigating the destructive role of perturbations induced on planetesimal disks by wide binary companions (Rafikov 2013; Rafikov & Silsbee 2015; Silsbee & Rafikov 2015; Sefilian 2017).

We exploit that feature here by appealing to the negative precession induced by an extended and massive trans-Neptunian debris disk to mitigate against—and, if possible, freeze—the prograde differential precession induced by the outer planets on a distinguished population of TNOs that is yet to be identified.

With this in mind, we recover the secular orbit-averaged disturbing potential, R_d_, of power-law disks up to fourth order in the orbital eccentricity of a coplanar test particle (see Appendix A):

(4) $$R_{d}=K\left[\psi_{1}e_{p}\cos∆\varpi +\psi_{2}e_{p}^{2}+\psi_{3}e_{p}^{2}\cos\left(2∆\varpi \right)+\psi_{4}e_{p}^{3}\cos∆\varpi +\psi_{5}e_{p}^{4}\right],$$

where

(5a) $$K=\pi G\sum_{0}a_{\mathrm{out}}^{p}a_{p}^{1-p}>0,$$

(5b) $$∆\varpi \equiv \varpi_{p}-\varpi_{d}.$$

The dimensionless coefficients *ψ*_i_ are given by Equations (25)–(30).

Orbit-averaged quadrupolar action of the outer planets is captured via

(6) $$R_{p}=+\frac{1}{3}\Gamma {\left(1-e_{p}^{2}\right)}^{-\frac{3}{2}},$$

with

(7) $$\Gamma =\frac{3}{4}\frac{GM_{⊙}}{a_{p}}\sum_{i=1}^{4}\;\frac{m_{i}a_{i}^{2}}{M_{⊙}a_{p}^{2}},$$

and [(*m_i_*, *a_i_*) = 1̤4] the masses and current semimajor axes of the four giant planets.

Combining both contributions, Hamilton’s equations for the signed “angular” momentum $l_{p}=\pm \sqrt{1-e_{p}^{2}}$ and the conjugate longitude of the apse ϖ_p_ are given by:

(8) $$L_{p}l_{p}=-K\left[\mathrm{sin\Delta}\varpi \left[\psi_{1}\sqrt{1-l_{p}^{2}}+\psi_{4}{\left(1-l_{p}^{2}\right)}^{\frac{3}{2}}\right]+2\psi_{3}\left(1-l_{p}^{2}\right)\sin(2\Delta \varpi )\right],$$

and

(9) $$L_{p}{\dot{\varpi }}_{p}=\frac{\Gamma }{l_{p}^{4}}+Kl_{p}\left[\frac{\psi_{1}\mathrm{cos\Delta}\varpi }{\sqrt{1-l_{p}^{2}}}+2\psi_{2}+2\psi_{3}\cos(2\Delta \varpi )\right.\left.+3\psi_{4}\mathrm{cos\Delta}\varpi \sqrt{1-l_{p}^{2}}+4\psi_{5}\left(1-l_{p}^{2}\right)\right],$$

with

$L_{p}=\sqrt{GM_{⊙}a_{p}}$, the constant angular momentum conjugate to the mean anomaly, which has been averaged out of the game. Disturbing functions (Equations (4), (6)), and equations of motion (Equations (8), (9)) govern the dynamics of both prograde and retrograde orbits, which are coplanar with disk and planets. Below, and in keeping with observed aligned TNOs, we concentrate primarily on the prograde phase space.

**Figure 1:**
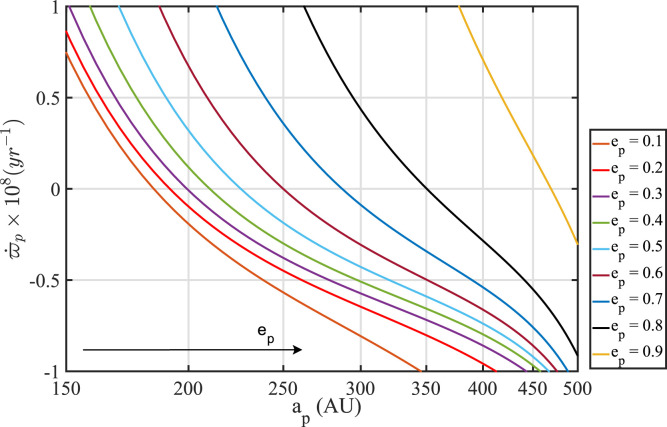
Rate of apse precession ϖ_p_ of TNOs that are antialigned with the disk (Δϖ = π), over a range of semimajor axes *a_p_* for different values of eccentricity ep. Precession is here driven by the combined action of the giant planets and the fiducial disk model DM1 (see Table 1). Zero apse-precession is obtained for all the considered values of *e_p_*, and semimajor axes ap between 150 and 500 au. Given that the torque vanishes for Δϖ = π (see Equation (8)), we have here a family of stationary orbits that are antialigned with the disk and whose eccentricity grows with *a_p_*.

In Figure 1, we display the apsidal precession rate induced by the outer planets and the fiducial power-law disk model (Table 1, model DM1) on orbits that are antialigned with the disk’s spatial orientation (i.e., orbits with Δϖ = π), over a range of semimajor axis a_p_, and for different values of TNO eccentricity *e_p_*. Evidently, there is an eccentric antialigned orbit with zero net apse precession at all semimajor axes in the considered range. Keeping in mind that the torque (Equation (8)) is null for Δϖ = π, we have here evidence for a one-parameter family of antialigned stationary orbits that will provide the skeletal structure around which the observed TNOs—and the rest of our paper—will be fleshed out.

**Figure 2:**
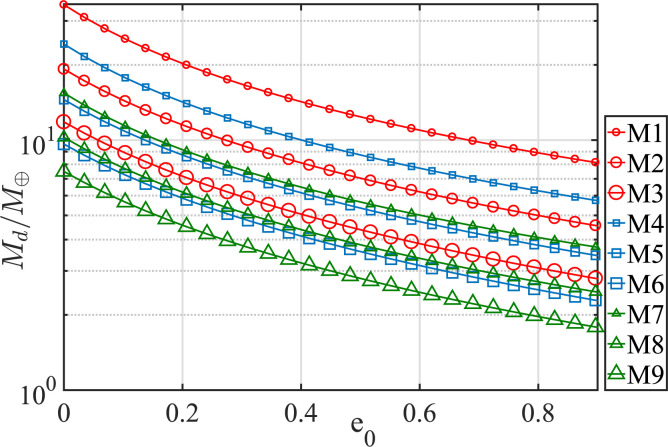
The required disk mass *M_d_* as a function of disk eccentricity (*q* = 0, *e_d_* (*a_p_*) = *e_0_*) to obtain stationary antialigned (Δϖ = π) TNO orbits at *a_p_* = 257 au with *e_p_* = 0.82. The calculation is performed for disk parameters (*p, a*_i_n and *a*_out_) given in Table 2. When combined with results shown in Figure 1, this figure speaks for the robustness of our proposed mechanism.

Before we examine the full dynamical behavior of this family, we think it reasonable to probe the robustness of this remarkable, broad-ranged cancellation of apse-precession to variations in disk properties (mass density profile, disk eccentricity, disk radial extent). We thus computed the disk mass M_d_, which is required to apse-freeze an antialigned orbit (Δϖ = π) of given eccentricity *e_p_* and semimajor axis a_p_ when embedded in a disk of given mass distribution (dictated by p), inner and outer edge, and e_0_. The outcome of this exercise for a test particle with *a_p_* = 257 au and *e*_*p*_ = 0.82 is shown in Figure 2, and permits the following conclusions: (a) the required disk mass can be as low as ∼1 *M*_⊕_ and as high as ∼30 *M*_⊕_; (b) lower M_d_ is required at higher disk eccentricity, an effect that is surely due to enhancement of disk- induced retrograde precession with increasing e_0_; (c) the critical M_d_ increases with increasing a_out_. This behavior is evident in axisymmetric disks where the disk induced precession is well- approximated by the following expression^3^

(10) $${\left.{\dot{\varpi }}_{p}\right|}_{\mathrm{disk}\;}≃-4.2\times 10^{-10}{yr}^{-1}\frac{M_{d}}{1M_{⊕}}\frac{10^{3}au}{a_{\mathrm{out}\;}}{\left(\frac{a_{p}}{500au}\right)}^{-0.5}$$

for circular TNO orbits. What is evident for axisymmetric disks is clearly maintained in eccentric ones. Furthermore, we checked that our conclusions for a single antialigned equilibrium orbit (with *e_p_* = 0.82 and *a_p_* = 257 au) hold for all.

Exhaustive exploration of the dynamics sustained by our orbit-averaged Hamiltonian shows that the fiducial disk model (Table 1, DM1) harbors three distinct families of orbits:

1. A family of stable, highly eccentric, and antialigned orbits (Δϖ = π): this family shows equilibrium *e_p_*(*a_p_*)-behavior that is remarkably consistent with the trend followed by clustered TNOs. It is the family of most interest to us in relation to the shepherding phenomenon.
2. A family of stable, aligned (Δϖ = 0), and low-eccentricity orbits: interestingly enough, this family follows in its trend the eccentricity distribution of the disk that hosts it.
3. A family of highly eccentric and aligned orbits (Δϖ = 0): this family parallels the behavior of the stable, high *e_p_*, antialigned family, but is doomed to instability.

**Figure 3:**
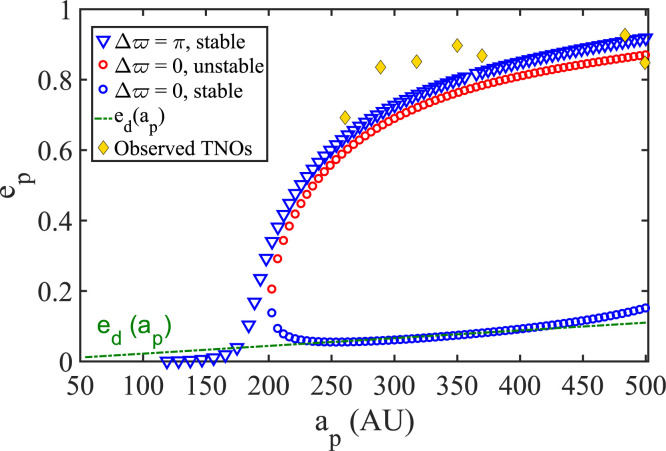
The stationary TNO families (*e_p_*, Δϖ), both stable and unstable, that are sustained by DM1 (Table 1) acting together with the giant planets. The stable antialigned (Δϖ = π) family follows quite closely the observed *e_p_* - *a_p_* trend of the seven clustered TNOs that are considered in this study (Table 3). A stable family of nonprecessing orbits that are aligned with the disk (Δϖ = 0) has an eccentricity profile that is almost identical to the imposed disk eccentricity profile *e_d_* (*a_p_*).

Taking it for granted that the stable antialigned family correlates with the observed family of clustered TNOs, we conclude that DM1 naturally excludes stable high-eccentricity clustering in the opposite apse orientation, an orientation where significant high-eccentricity clustering is apparently not observed.^4^ All three families are shown in Figure 3, together with the eccentricity distribution of the underlying disk.

**Figure 4:**
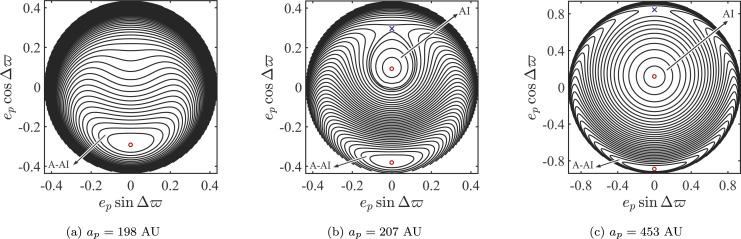
Phase portraits corresponding to the total Hamiltonian, *H_p_*+ *H_d_*= -(*R_p_*+ *R_d_*), in *e_p_*(sin Δϖ, cos Δϖ) space, at three different TNO semimajor axes *a_p_* for the fiducial disk model DM1. The semimajor axes were chosen to illustrate the existence and bifurcation of the families identified in Figure 3. The stable (unstable) secular equilibria are highlighted in red (blue). Panel (a) shows the phase portrait at *a_p_* = 198 au with a single stable antialigned equilibrium and its associated A-AI (Anti-Aligned Island) situated at Δϖ = π. In panels (b) and (c), we show the phase portrait at *a_p_* = 207 au and *a_p_* = 453 au, respectively, with two new aligned equilibria (Δϖ = 0), one unstable and one stable, the latter coming with an AI (Aligned Island) of librating orbits. The *e_p_*-*a_p_* trends of Figure 3 are evident with the progression in semimajor axis through panels (a), (b), and (c), respectively.

These families can be further situated within the global phase space structure that is captured in Figure 4 at three distinct semimajor axes. In addition to equilibria and their bifurcations, the phase diagrams reveal aligned and antialigned islands (AI and A-AI respectively) of bounded motion around the parent stable equilibrium orbits. These islands host orbits that will show signs of clustering (aligned and antialigned, respectively) when considered collectively and in time. The A-AI shelters high-eccentricity orbits that straddle, as they oscillate in their eccentricity and longitude of apse, the parent equilibrium family that so closely follows the *e_p_*–a_p_ trend of the observed TNOs. We will have more to say about this population when we discuss it in its full 3D glory below. The AI, on the other hand, is populated by orbits that share, on average, the orientation and orbital eccentricity of the host disk, thus providing a rich supply of orbits with which to construct a self-consistent deformation of DM1 (an exercise on which we comment in Section 5). It further includes orbits that have large amplitude eccentricity variations that bring them close to the unstable high-eccentricity aligned orbits. Such orbits tend to linger around that unstable aligned configuration, projecting a temporary sense of eccentric alignment with the disk, which is then lost to evolution on timescales that are long enough.^5^

The AI and A-AI are both surrounded by high-eccentricity orbits that circulate in the longitude of the apse while maintaining large and near-constant eccentricity. We shall say more about these populations when we discuss curious members of the TNO population in Section 5.

In sum, DM1 shepherds eccentric antialigned orbits (Δϖ = π) whose properties favor them as coplanar analogs of the family identified by Trujillo & Sheppard (2014), while at the same time supporting aligned and nonprecessing orbits of moderate eccentricity, which promise to reproduce the disk supporting them, in a self-consistent treatment of the dynamics.^6^ It would thus seem that a massive eccentric trans-Neptunian debris disk, together with the action of the outer planets, provides significant and profoundly suggestive clustering of embedded test particles. Whether such a disk obviates the need for a Planet Nine-like perturber altogether will be discussed further below after we explore out-of-plane dynamics, close to where the observed TNOs tend to roam. However, what is already clear at this coplanar stage is that the action of such a potential disk (which is evidently felt by highly eccentric orbits for disks with mass as low as ∼1 *M*_⊕_) cannot be ignored, and will have to be considered together with any putative extra planet.

## 3. Life Outside the Plane: Going 3D

Freezing coplanar orbits is interesting enough. However, the observed TNO bunch is held together in inclined orbits. Can we say anything about inclinations? There is, in principle, no hurdle to generalizing the proposed mechanism to inclined orbits. However, an attempt to work it out with our orbit- averaged treatment of a razor-thin disk potential faces an insurmountable singularity (Heppenheimer 1980). Disk height comes in to the rescue, but then expressions become arbitrary without a specific prescription for vertical disk structure (Hahn 2003). One fix is to use a local approximation in the averages, which regularizes expressions (Ward 1981) and allows us to explore eccentricity-inclination dynamics for both axisymmetric and eccentric disks—but we went further.

Starting with the mass and eccentricity distributions in DM1, we computed the full 3D potential and recovered associated spherical harmonics numerically (M. Kazandjian et al. 2018, in preparation). We then orbit-averaged spherical harmonics (again numerically) and obtained closed-form expressions for any given semimajor axis, to the desired (arbitrary) order in eccentricity and inclination. A brief explanation of the steps involved is provided in Appendix B.

With the orbit-averaged mean field of the razor-thin eccentric disk in hand (Equation (39)), we added the secular contribution of the outer planets (Equation (41)) to study the coupled eccentricity-inclination dynamics of a particle in a perfectly smooth fashion. With the help of these expansions, we could study off-plane dynamics of TNOs that are clustered in the plane, and determine stability to small inclinations, as well as the long-term evolution of populations of initially clustered and inclined objects. A brief report on the global dynamics follows:

1. As evident in Figure 15, planar phase-space structure (including families of equilibria, their stability, and their behavior as a function of semimajor axis) is recovered quite accurately within this generalized formalism;
2. An involved *linear* stability analysis confirmed that families of stable planar eccentric equilibria (both aligned and antialigned with the disk’s apsidal line) are further stable to small perturbations in inclination: this is quite encouraging because it suggests that the flock of stationary orbits that were identified in the plane is maintained when subject to small out-of-plane perturbations.
3. Small-amplitude variations in the inclinations, eccentri- cities, and longitude of apse around stable coplanar equilibria were numerically shown to maintain *near alignment* in the longitude of apse, all the while the argument of apse and longitude of node *circulate*.
4. Moving to large amplitude variations in inclination: Any temptation to inquire about fixed antialigned, eccentric and sufficiently inclined orbits is quashed by the realization that both inner quadrupole and eccentric disk induce retrograde nodal precession. While varying with location in and/or inclination to the disk, the reinforced retrograde precession excludes the possibility of apse-aligned orbits that further share the same spatial orientation.

We could, of course, proceed to provide a complete classification of orbital dynamics in the combined field of disk and planets. This is a two-degree-of-freedom problem that is amenable to description in terms of Poincaré sections at any given semimajor axis. We think that such an exercise is best relegated to a separate purely dynamical treatment. Instead, we opt to follow populations of judiciously chosen particles over the underlying complex phase space, with a view to characterizing the extent to which our setup can reproduce observed metrics.

### 3.1. Populations over Phase Space

The reference disk, together with the outer planets, sustains two families of stable coplanar equilibria, one aligned (Δϖ = 0) and of low eccentricity, the other antialigned (Δϖ = π) and of large eccentricity. Antialigned equilibria follow the observed trend of eccentricity with semimajor axis (see Figure 3). It is natural to ask what remains of this trend when vertical heating is included, and when eccentricity-inclination dynamics kicks in. We spoke previously of linear stability of planar equilibria to slight inclination change. We have further reported results of numerical simulations showing that the long-term evolution of perturbed planar orbits, shows stability in inclinations for small enough inclination, while maintaining *confinement in the longitude of the apse*. That would suggest that populations of particles initiated around the islands of planar stability would maintain the planar alignment, though it is not immediately clear for how long in the presence of nonlinearities.

With this in mind, we explored the dynamics of populations of particles in the combined orbit-averaged gravitational field of DM1 and the outer planets over the age of the solar system. Particles were initiated around the AI and A-AI of stable planar equilibria (see Figure 4) at the semimajor axis locations of seven of the clustered objects listed in Table 3.^7^ Islands of stability were sampled uniformly in eccentricity, with inclinations assigned uniformly in a 10^ΰ^ range. The argument of pericenter ω_p_ and the longitude of ascending node Ω_p_ were picked to guarantee uniform Δϖ sampling in the range 180° ± 20° for the antialigned family, and?±20° for the aligned family. This way, we end up with 300 particles at each of the seven observed semimajor axes, and follow their orbits over the age of the solar system.

**Table 2:** Power-law Disks Used to Generate Figure 2

Model	*a_in_*(au)	*a_out_*(au)	^p^
M1	200	1200	0.1
M2	…	…	0.5
M3	…	…	0.9
M4	200	1000	0.1
M5	…	…	0.5
M6	…	…	0.9
M7	200	800	0.1
M8	…	…	0.5
M9	…	…	0.9

**Note.**We have adopted a constant disk eccentricity by setting *q* = 0, 0 < *e_d_*(a_p_) = *e_o_ <* 0.90.

**Table 3:** Heliocentric Semi Major axis (*a*_*p*_), Perihelion Distance (*q*_*p*_), Inclination (*i_p_*), Argument of Perihelion (ϖ_*p*_), Longitude of Ascending Node (Ω_*p*_), and Longitude of Perihelion (ϖ_p_) of the Clustered TNOs with *a_p_* > 250 au and *q_p_* > 30 au Considered in This Study

TNO	*a_p_* (au)	*q_p_* (au)	*i_p_* (°)	*ϖ_p_* (°)	*Ω_p_* (°)	*ϖ_p_* (°)
2012 VP_113_	260.8	80.3	24.1	292.8	90.8	23.6
2014 SR_349_	289.0	47.6	18.0	341.4	34.8	376.2
2004 VN_112_	317.7	47.3	25.6	327.1	66.0	33.1
2013 RF_98_	350.0	36.1	29.6	311.8	67.6	379.4
2010 GB_174_	369.7	48.8	21.5	347.8	130.6	118.4
2007 TG_422_	483.5	35.6	18.6	285.7	112.9	38.6
Sedna	499.4	76.0	11.9	311.5	144.5	96.0

**Note.** Data obtained from the minor planet center.

We characterize an orbit’s orientation and eccentricity with the Lenz

(11) $$e_{p}=e_{p}\left(\begin{array}{c} \cos\omega_{p}{\mathrm{cos\Omega}}_{p}-\cos i_{p}\sin\omega_{p}{\mathrm{sin\Omega}}_{p}\\ \cos\omega_{p}{\mathrm{sin\Omega}}_{p}-\cos i_{p}\sin\omega_{p}{\mathrm{cos\Omega}}_{p}\\ \sin i_{p}\sin\omega_{p} \end{array}\right)=e_{p}\hat{e}$$

and specific angular momentum

(12) $$h=\sqrt{GM_{\Theta }a_{p}\left(1-e_{p}^{2}\right)}\left(\begin{array}{c} \sin i_{p}{\mathrm{sin\Omega}}_{p}\\ -\sin i_{p}{\mathrm{cos\Omega}}_{p}\\ \cos i_{p} \end{array}\right)$$

vectors. The Lenz vector lives in the plane of a particle’s orbit and points to its periapse; the angular momentum vector is perpendicular to the orbital plane, and its dynamics encodes nutation and precession of the orbit. Orbits can have aligned Lenz vectors while being spread out in node and inclination. Alternatively, they can be spread out in Lenz vector while sharing the same orbital plane. In other words, the behavior of both vectors must be known in order to completely characterize the degree of spatial alignment of a population of orbits, with the following metrics being particularly useful in that regard (Millholland & Laughlin 2017):

1. The departure of Lenz vector orientation from the mean as captured by
  (13) $$S_{\varpi }\left(t\right)=\sum_{i=1}^{7}e_{m}\left(t\right).{\hat{e}}_{i}\left(t\right),$$
  where *e^* (t), *i* = 1 ,…, 7] are unit Lenz vectors and *e_m_*(*t*) is their mean unit vector. This definition of *S*_ϖ_(*t*) allows us to quantify the degree to which the Lenz vectors are clustered about their mean. Specifically, if the Lenz vectors of the seven objects coincide with their mean at a given time, then *S*_*ϖ*_ (*t*) t = 7
2. A measure of the antialignment with respect to the trans-Neptunian disk as given by
  (14) $$A_{\varpi }\left(t\right)=e_{m}\left(t\right).{\hat{e}}_{d},$$
  where the disk orientation is fixed such that *e^_d_* = (cos ϖ_d_, sin ϖ_d_, 0)|_ϖd = π_ unless otherwise stated. A measure of *A*_ϖ_(*t*) = -1(+1) corresponds to configurations where the mean Lenz unit vector of the seven bodies is perfectly antialigned (aligned) with that of the disk at a given time.
3. A measure of clustering in ϖ_p_, ω_p_, and Ω_p_ separately as provided by the mean over unit vectors; *r*_ϖp_, *r*_ωp_ that circulate with these angles, respectively. The associated vectors have zero mean when they are homogeneously distributed on a circle, while perfect alignment results in their mean having unit length.

The objects of interest to us, listed in Table 3, yield a current value of *S*_ϖ_ » and A_ϖ_» -0.77, assuming a disk whose apsidal angle is 180^°^ away from the mean of the clustered inclined bunch. Moreover, the measures of *r* for the group of clustered objects are *r*_ω_ = 0.93, *r*_Ωp_ = 0.81 and *r_ϖp_* = 0.80, indicating confinement in both ω*_p_* and Ω_p_ as noted by Batygin & Brown (2016).

**Figure 5:**
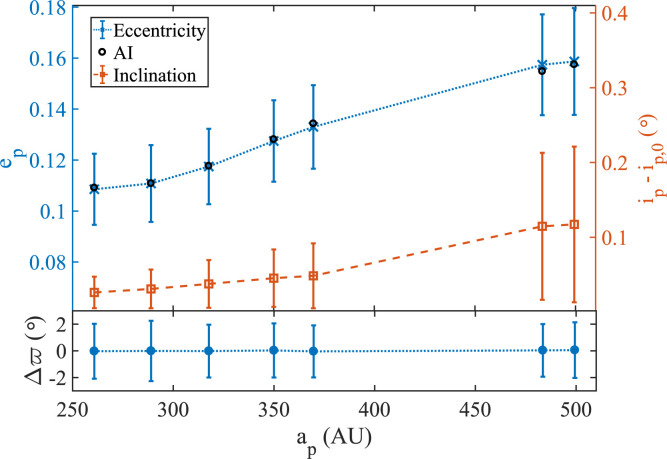
The average behavior of eccentricity, inclination, and longitude of periapse, and excursions thereabout, for a flock of objects initiated in the neighborhood of the AI. The top panel shows particles at all semimajor axes executing slight excursions in eccentricity and inclinations: values of *e_p_* remain close to the planar equilibria while inclinations oscillate around the initial conditions *i_p,0_*. The bottom panel shows how all these particles maintain alignment with the disk with Δϖ ≈ 0°., with negligible spread, over the relevant range in *a^p^*.

**Figure 6:**
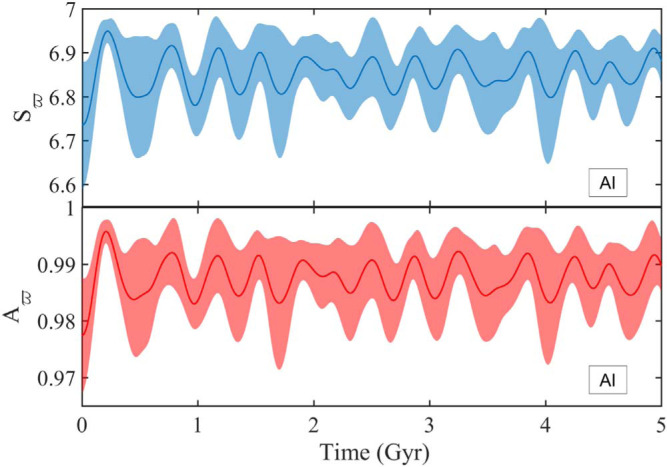
Time evolution of *S_ϖ_* and *A_ϖ_* for objects initially near the AI. The calculation is based on an ensemble of 10 sets of particles, each set consisting of seven particles randomly picked within the AI at each of the considered semimajor axes. The thick lines represent the ensemble-averaged values and the shaded regions enclose the spread around the average.

**Figure 7:**
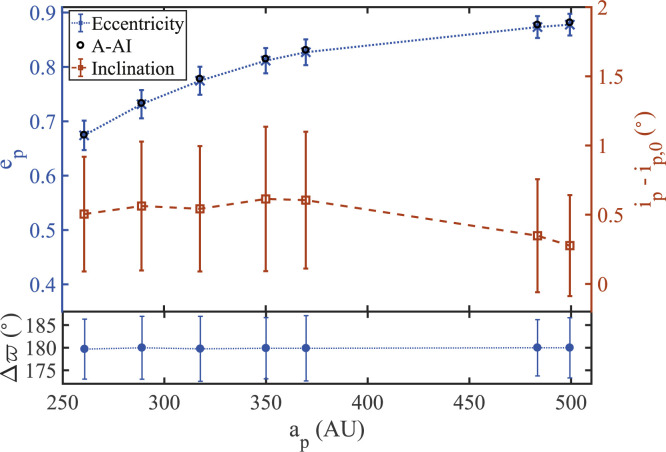
Average values of eccentricity, inclination, and longitude of periapse, and spread thereabout, for objects initiated near the A-AI with initial inclinations *i_p_*,0 < 5°. It is evident that slightly inclined particles initiated around the A-AI exhibit an average behavior of *e_p_*-*a_p_* consistent with the planar equilibrium profile. At the same time, as shown in the bottom panel, the orbits remain clustered in ω and antialigned with the disk, with Δω ~ 180° at all *a^p^*.

Based on extensive orbit integrations of our samples, we learned the following:

1. Particles initiated around the AI (Figure 4 (b), (c)) stay tightly bunched while showing small amplitude varia- tions in their inclination, eccentricity, and longitude of apse (see Figure 5). Indeed, we find a time-averaged value of *r_ϖp_* =0.989 ± 0.011. indicating strong ϖ_p_-confinement that is maintained by the opposite circulation of ω_p_ and Ω_p_ as reflected in *_Ωp_* =0.040 ± 0.003. In other words, the orbit structure that is expected to self-consistently reproduce the planar disk is stable enough to inclined motion to hold the promise of sustaining a thick version of that disk. In Figure 6, we show ensemble-averaged behavior of *A_ϖ_*(*t*) and *S_ϖ_*(*t*), which supports the conclusion above, with the time-averaged *S_ϖ_* ~ 6.85 and *A_ϖ_* ~ 0.98 indicating Lenz vector confinement as well as disk alignment in the neighborhood of the AI.
2. Populations of particles initiated around the A-AI (Figure 4(b)) show greater complexity as a function of semimajor axis and inclination. Particles with semimajor axes 250 ⪯ *a_p_* ⪯ 350 au librate around the planar island with slight excursion in inclination and eccentricity for all initial inclinations (see Figure 7): linear stability translates into long-term stability in this case, with low- inclination orbits maintaining Lenz vector alignment all the while displaying a spread in node and periapse. Indeed, our simulations show that around 93% of all the considered orbits with *a_p_* ⪯ 350 au are strongly confined in ϖ_p_ with *r_ϖp_* > 0.9 while the periapse and node circulate (*r_ωp_* ≤ 0.05 and *r_Ωp_* ≤ 0.03). On the other hand, particles with *a_p_* $\underset{\sim }{≻}$ 350 au show long-term behavior that depends on the initial inclination: (i) Particles launched with *i_p_* $\underset{\sim }{≻}$ 4° - 5° are stable to off-plane motion, similar to the objects at *a_p_* ≤ 350 au; (ii) In contrast, particles launched with *_p_* > 5° show large amplitude variations in *e_p_* and *i_p_*, with inclinations growing somewhat erratically to values higher than 20^°^, all the while *e_p_* evolves to smaller values. Such particles still show significant clustering in ϖ_*p*_ with *r_ϖp_* ~ 0.6, though weaker than what is observed with stable inclined populations.

**Figure 8:**
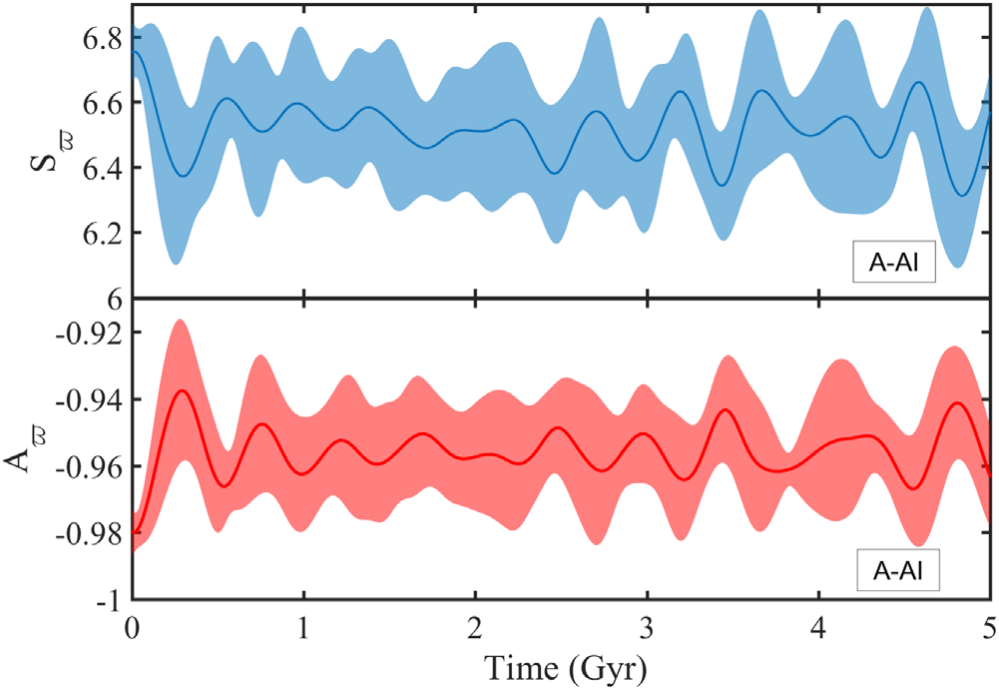
Time evolution of the ensemble-averaged *S_ϖ_* and *A_ϖ_* (thick lines) and the spread thereabout (shaded regions) for objects sampled near the A-AI, with *i_p_* (*t* = 0) < 5°. Clustering of Lenz vectors is maintained at all times (*S_ϖ_*(*t*) ~ 6.5) together with disk antialignment (*A_ϖ_*(*t*) ~ -0.96).

As evident in Figures 7 and 8, particles initiated with low inclinations (*i_p_* < 5°) around the A-AI remain clustered and antialigned with the disk, with small amplitude variations in their eccentricities and inclinations. Their stability guarantees the survival of a puffed-up version of the backbone of coplanar antialigned equilibria (Δϖ = π) whose *e_p_*-a_p_ behavior is consistent with the observed TNOs.

### 3.2. Clones of Observed TNOs

We probe the orbital evolution of “clones” of observed TNOs (Table 3) over the age of the solar system. At each of the considered semimajor axes, we build samples of 300 particles with orbital elements randomly picked in the neighborhood of the observed ones (*δe* = 1%*e*_obs_ and *δi* = *δω* = *δΩ* = 5°). The disk is again coplanar with the outer planets and antialigned with the mean apse direction of the clustered TNOs.

**Figure 9:**
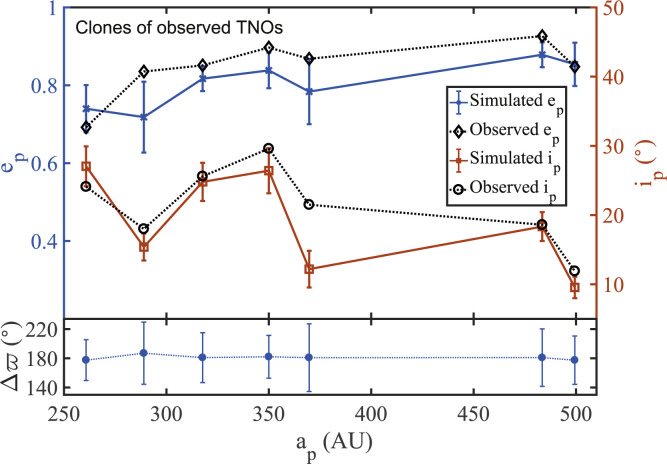
The average eccentricity, inclination, and longitude of apse along with their time-averaged spread for all the “successful clones” in our simulations. The top panel reveals reasonable agreement between observed and average values of both *e_p_* and *i_p_*. The bottom panel indicates that clustering and antialignment with the proposed disk (DM1) are maintained at all considered values of *a^p^*.

We find that more than 60% of the clones maintain, at all times, a perihelion distance larger than the orbital radius of Neptune. We dub these objects “successful clones” (SCs for short) and analyze their orbital evolution to conclude that:

1. SCs follow quite closely the eccentricity and inclination of their progenitors (see Figure 9);
2. SCs, on average, maintain antialignment with the disk apsidal line, while showing slight oscillations in the longitude of apse around the mean; see Figure 9. Considering all successful simulations, we find *r_ϖp_* » 0.785, which compares well with that of the observed bunch; *r_ωp_* = 0.80;
3. SCs show no confinement in Ω_*p*_ and ω_*p*_, with *r_Ωp_* = 0.020 ± 0.006 and *r_ωp_* = 0.033 ± 0.019.

**Figure 10:**
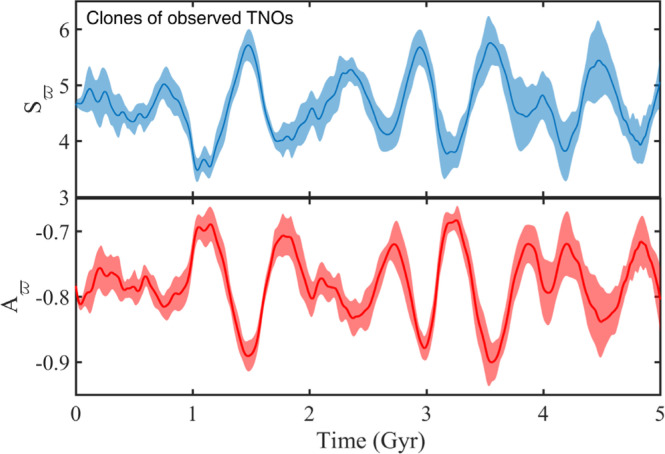
Evolution of both *S_ϖ_* and *A_ϖ_* as a function of time for clones of the observed TNOs. The calculation is done with ten ensembles of seven particles each, randomly picked from the population of SCs at each of the seven considered values of *a^p^*. Thick lines represent the mean; shaded regions indicate the spread about that mean. *S_v_*(*t*) and *A_v_*(*t*) oscillate around their initial values, indicating vp-confinement that, on average, is 180. away from the fixed disk *a^p^*sidal line.

Computing *A_ϖ_* (*t*) and *S_ϖ_* (*t*) as before, except with ensembles of SCs, we recover *A_ϖ_* » -0.78 ± 0.03 and *S_ϖ_* » 4.63 ± 0.34 (refer to Figure 10 for the full behavior of both metrics). The latter is in agreement with *S*_ϖ_(*t* = 0) » 4.57 for the observed TNOs, while the former is consistent with the expected value of *A*_ϖ_(*t* = 0) ≈ -0.77, assuming that the mean apsidal angle of the observed TNOs is 180^°^ away from that of the hypothesized disk.

In short, the simulations we carried out show that the envisioned disk of trans-Neptunian icy bodies (DM1, to be specific) can provide a fair amount of ϖ-confinement for particles whose orbits are seeded in the neighborhood of the observed clustered TNOs.

## 4 Variations on a Theme

Given uncertainties about disk mass, eccentricity, self- consistent precession, etc., we thought it reasonable to explore a range of disk properties around the fiducial ones adopted in what preceded. Below is a brief account of what we learned, supplemented by an appropriate figure, when called for. These variations will be assessed with observed properties and disk self-consistency in mind. They will serve to inform our discussion of how a disk of the desired properties forms in the first place.

**Figure 11:**
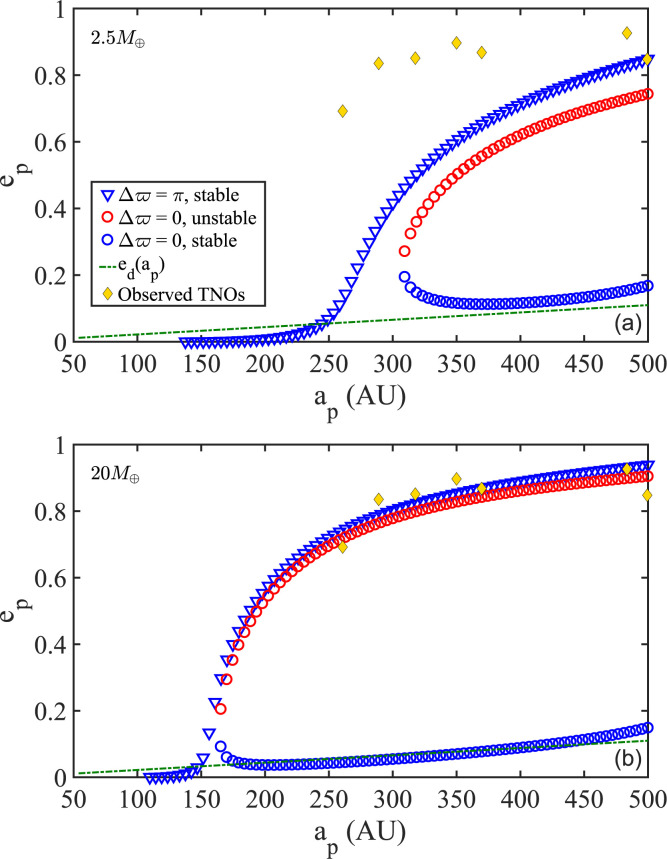
Coplanar families of stable and unstable equilibria sustained by disk models DM2 and DM3 (see Table 1). DM2 and DM3 are identical to the fiducial DM1, except that DM2 is less massive with 2.5 MÅ (panel a) and DM3 more massive with 20 MÅ (panel b). Evidently, increasing Md drives up the equilibrium *e_p_* while maintaining the stability of the three families, in agreement with our expectation (see Equation (15)). Furthermore, and as evident in panel b, massive disks provide a supply of aligned orbits with *e_p_* ~ *e_d_* over a broader range of *a^p^*.

**Disk Mass**. More massive disks, all else being kept the same, maintain planar equilibria of higher eccentricity—which, as is clear by now, will carry over to properties of spatially aligned populations. Figure 11 illustrates the effect in disks that are less and more massive than the adopted reference disk (DM2 and DM3 in Table 1). This behavior is not too difficult to recover from model Equations (8) and (9), which reduce to

(15) $$e_{p}≃{\left[1-c\times M_{d}^{-\frac{2}{5}}\times a_{p}^{\frac{2}{5}(p-4)}\right]}^{0.5}$$

under the assumption of axisymmetry (*e_d_* 0) where the constant $c=f\left(p\right)\times {\left(a_{\mathrm{out}}^{2-p}\sum_{i=1}^{4}m_{i}a_{i}^{2}\right)}^{\frac{2}{5}}>0$.^8^

Thus, a better fit with the observed eccentricities (ignoring eccentricity-inclination dynamics) can be achieved with a disk mass higher than the one adopted in our analysis (see Figure 11(b)). Furthermore, the bifurcation of equilibria into aligned and antialigned families is seeded earlier in a massive disk, such as DM3, implying that such disks will have a supply of aligned orbits (Δ*ϖ* = 0) over a broader range of semimajor axes with which to build themselves up. If explanation is required, the reader should keep in mind that a more massive disk allows for stronger precession, hence the ability of low- eccentricity orbits to withstand the differential precession induced by the planets at smaller semimajor axes than otherwise possible with a lighter disk; see Figure 11.

**Figure 12:**
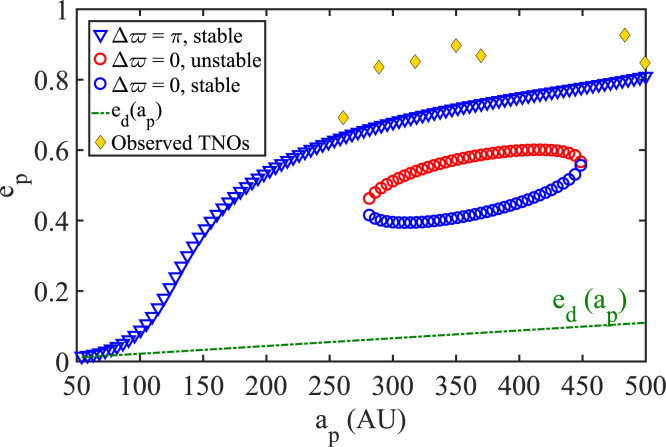
The equilibrium TNO families sustained by a disk model analogous to DM1, but with more mass concentrated in the inner parts, *p* = 2.5 (DM4 in Table 1). It is evident that, although the assumed disk model gives rise to a coplanar stable family of antialigned high *e_p_* orbits, such a disk cannot harbor TNO orbits aligned with the disk such that *e_p_* ~ *e_d_* (*a_p_*). Such is the case in all disks with mass dropping outward (p < 2).

Thus far, we have taken for granted disk models that have mass increasing with *a_p_*. We now ask how things would differ in a disk with mass dropping outward. As evident in Figure 12, in a disk, which is otherwise analogous to the fiducial model, but with *p* = 2.5 (DM4 in Table 1), it appears impossible to support orbits that are aligned with the disk and have *e_p_* ~ *e_d_*(*a_p_*). Such (apse-aligned) disks appear unable to sustain themselves and will not be discussed any further.

**Disk Eccentricity**. Here, one can change both the eccentricity profile (via *q*) and the eccentricity at the outer edge of the disk (via *e*_0_), for a given profile. Reporting on our thorough exploration of the rich set of bifurcations that are obtained as a function of disk eccentricity and their implication for the structure of the disk itself would take us too far afield. Suffice it to say that increasing the outer-edge eccentricity in negative-q disks (i.e., *de_d_/da_d_* > 0) or adopting eccentricity profiles that drop outward, keeping all else invariant, introduces greater complexity in the structure of both antialigned and aligned planar equilibria, but unfortunately at the cost of losing those disk-aligned orbits that we believe will be essential in any self-consistent reconstruction of an eccentric disk. We find that, for disks structured such that the bulk of their mass is in the outer parts, a disk eccentricity of *e_0_* » 0.20-0.25 (with negative *q*) is the *maximum* that can be tolerated before the eccentricity behavior of the disk-aligned family no longer follows that of the underlying disk.

**Disk Precession**. A self-gravitating eccentric disk, which is further torqued by the outer planets, is likely to precess as a whole. The actual rate of precession of a saturated, nonlinear, eccentric mode is difficult to ascertain. The timescale associated with self-sustained precession is on the order of the secular timescale in the disk, ≃(*M_⊙_*/*M_d_* × *T_Kepler_*, and comes out to ≃10^10^ yr for a circular TNO orbit in a 1 *M*_⊕_ axisymmetric trans-Neptunian disk.^9^

This is then superposed with differential precession induced by the outer planets, with a timescale of 10^10^ yr. Actually, we can write the contribution of the giant planets to the total TNO precession rate as

(16) $${\dot{\varpi }}_{p}{|}_{\mathrm{planets}}\approx +1.93\times 10^{-10}{yr}^{-1}{\left(\frac{500au}{a_{p}}\right)}^{3.5}$$

for circular TNO orbits.

**Figure 13:**
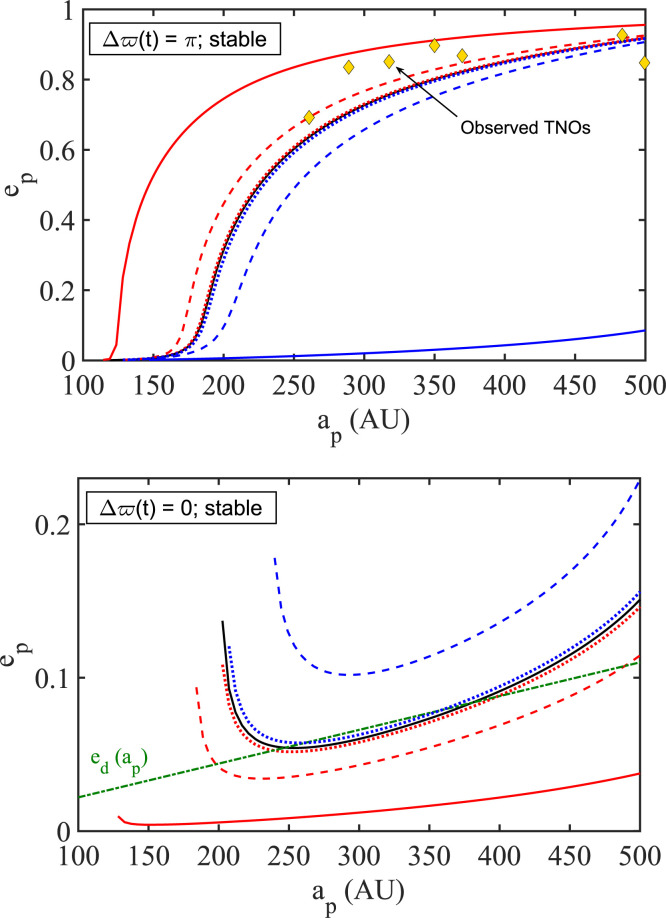
The effect of rigid disk precession, both retrograde (in blue) and prograde (in red), on the two coplanar stable equilibrium TNO families sustained by non-precessing DM1 (in black). Precession rates of increasing order, 0.02, 0.2, and 2 × 10^-8^ yr^-1^, are depicted by dotted, half-dashed, and full lines, respectively. The results show that prograde disk precession increases (lowers) the eccentricity of the high(low)-ep family; one has the opposite effect with ϖ_d_ < 0, and this with increasing severity for larger |ϖ|. For instance, equilibrium orbits aligned with the disk (ϖ_v_(*t*) = 0) are not sustained if ϖ_d_ = 2 × 10^-8^ yr^-1^. Note that the stability of each equilibrium family is maintained in precessing disks.

**Figure 15:**
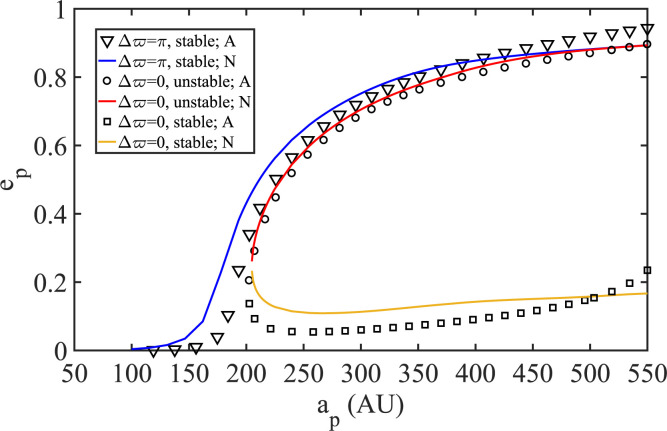
Comparison between the coplanar equilibrium families obtained via orbit-averaged disk potential (A; Equation (4)) and orbit-averaged harmonics (**N**; Equation (39) with *i_p_* = 0) of the potential generated by DM1 (Table 1). It is evident that both treatments of the disk potential, when combined with that of the giant planets, yield very similar equilibrium structure, including stability and behavior as a function of semimajor axis.

We explore the structure of equilibria in uniformly precessing disks at three progressively faster pattern speeds (both prograde and retrograde). The results are shown in Figure 13. For the disk mass being considered, it is evident that, with prograde precession, agreement with the observed eccentricity profile improves at the risk of losing dynamical support from the eccentric aligned and stable family of orbits which acquire lower values of *e_p_* with increasing ϖ_*d*_. On the other hand, retrograde precession worsens agreement with the observed family, while shifting the eccentricity profile of the aligned family to eccentricities that are too large to sustain the precessing disk.

That the desirable features of our fiducial disk are disturbed by imposed precession is, of course, not surprising; its properties were optimized under the assumption of zero precession. However, now that we have a sense of the effect of uniform precession, we can consider scenarios in which we optimize over disk properties and precession simultaneously. In particular, one can foresee a lower mass disk undergoing prograde precession while at the same time matching observed high-eccentricity orbits and sustaining a family of stable aligned orbits over a broader range of semimajor axes.

## 5 Discussion

Our proposition, with its pros and cons, is perhaps not as singular in the context of planetary system formation as the Planet Nine hypothesis. Still, the ingredients that go into it— the origin of the disk, its mass, and its eccentricity, as well as the self-consistent maintenance of the disk itself—require a closer look, which we attempt below.

**Disk Mass**. There is, to be sure, much uncertainty concerning the mass that lies beyond Neptune, let alone the question of eccentricity and self-organization of that mass. We require an eccentric, lopsided, equilibrium disk (precessing or not) of 10 Earth masses or so. Standard pictures allow for, at most, a few tenths of an Earth mass to be scattered in that region in a primordial disk of planetesimals (Gladman et al. 2011; Silsbee & Tremaine 2018).

Arguments that put a few tenths of an Earth mass in that region are either based on extrapolations of observed size distributions or on numerical simulations of a scattered disk that would invariably allow for such low amounts in that region. As noted in the text, such low masses can contribute to ϖ-confinement, but they make for eccentricities in disagreement with what is observed (at least in the coplanar, non-precessing disk case—see Figure 11(a)). The question is, however, how serious are these constraints? Well, the distributions themselves are poorly constrained in their tail, and the dynamical arguments constrained by primordial assumptions that may or may not be legitimate. There are, of course, alternatives considered in the literature. Hills (1981) envisions an intermediate zone between the Kuiper Belt and the Oort cloud that is expected to harbor up to a few tens of Earth masses. Then there are suggestions that massive planetesimal disks may be a natural outcome of planet formation processes (Kenyon & Bromley 2004; Carrera et al. 2017; Eriksson et al. 2018).

Most important for us, however, is the exercise of Hogg et al. (1991), who consider the question of hidden mass and its gravitational signature; they conclude that, by looking at planetary motion—and more importantly, at cometary orbits— it is not unreasonable to expect up to a hundred or so Earth masses in the region in question. The exercise has not been revisited since (S. Tremaine 2018, Private communication), though related questions were recently examined in relation to the Planet Nine hypothesis (Bailey et al. 2016; Fienga et al. 2016; Lai 2016).

With the above in mind, it would seem that a massive trans- Neptunian debris disk is not securely ruled out, hence our suggestion: rather than lump the perturbing mass in a 10 Earth mass planet, and then find a way to push it out on an inclined and eccentric orbit (Kenyon & Bromley 2016; Parker et al. 2017; Eriksson et al. 2018), allow for the less daring hypothesis of a distribution of coplanar TNOs with a total of *M*_⊕_ and see what it does for you.

Disk Eccentricity. Of course, the existence of eccentric particle distributions with inner quadrupolar forcing is not foreign to the solar system, with the ò ring of Uranus providing an early example of the type (Goldreich & Tremaine 1979). In that context, it was argued that self-gravity provides resistance to differential precession induced by the planet’s quadrupole to maintain an eccentric equilibrium configuration for the ring. Similar arguments in favor of self-gravitating eccentric distributions are brought to bear on the lopsided double nuclei of galaxies (Tremaine 1995; Peiris & Tremaine 2003). Such a mechanism may also structure eccentric circumbinary and/or circumprimary protoplanetary disks (Kley et al. 2008; Paardekooper et al. 2008; Meschiari 2012). Here, we give evidence for a family of aligned and moderately eccentric orbits that promises to self-consistently build the disk that maintains it. Thus, there is little that is unusual about an eccentric disk, though the details of its origin remain to be explored.

Origin. Observations of ring systems, extrasolar debris disks, and stellar disks, as well as theoretical models and associated simulations suggest that eccentric disks are ubiquitous, rather easy to stimulate, and apparently also easy to sustain (with and without inner quadrupolar forcing). There are as many propositions for the origin of self-gravitating eccentric disks as there are dynamicists working in various contexts and at various scales: perturbation by passing objects (Jacobs & Sellwood 2001); dynamical instabilities that afflict low angular momentum (perhaps counter-rotating distributions) (Touma 2002; Tremaine 2005; Kaur et al. 2018); and forcing by eccentric inner or outer binary companion (Kley et al. 2008; Paardekooper et al. 2008; Marzari et al. 2009; Meschiari 2012; Pelupessy & Zwart 2013).

In numerical experiments with an eccentric, self-gravitating, narrow ring-like disk, Madigan & McCourt (2016) noted an inclination instability that was accompanied with a pattern of alignment in argument of periapse. The authors took that as an indication that the process may underlie the inclination-eccentricity behavior of the observed clustered TNOs. Intriguing though the proposition maybe, it suffers from various limitations: (a) simulations do not allow for inner quadrupolar forcing by the planets, or earlier, their being embedded in a massive gaseous disk; (b) simulations are not pursued long enough to follow the unfolding of the instability and its eventual relaxation; (c) it is hard to imagine how to form a disk of the required mass in the envisioned hot kinematic state.

It is likely that inner quadrupolar forcing, if strong enough, can quench the inclination-eccentricity instability altogether, a suggestion that is motivated by a related effect in the Kozai– Lidov type instability.

As to the observed pattern of alignment in argument of periapse, it is surely a transient of an in-plane instability, which is expected for high-eccentricity disks of the type considered (Kaur et al. 2018) and ultimately relaxes into a lopsided, uniformly precessing state of lower mean eccentricity. Gauss wire numerical simulations (M. Kazandjian et al. 2018, in preparation) confirm our expectations, with the disk of Madigan & McCourt (2016) relaxing into a thick, lopsided, uniformly precessing configuration in the presence of outer- planet quadrupolar forcing and remaining axisymmetric when we allow for outer planets that are ten times more massive.

Thus, while we believe the clustering mechanism of Madigan & McCourt (2016) is simply a transient that dissolves in time, it seems to do so in just the right sort of self- gravitating, eccentric, thick and uniformly precessing disk that, in combination with the outer planets, is expected to sustain antialigned orbits with behavior comparable to what is observed. The difficulty, of course, is that the initial conditions for the required instability (bias toward low angular momen- tum, highly eccentric orbits) seem far from what is expected of distributions of planetesimals at formation. This mechanism may be promising in its ultimate state, but it is somewhat unlikely in its origin.

**Comment on Disk Self-consistency**. Our proposition is predicated on the properties of an idealized disk and its gravitational impact on test particles that are embedded within it. We have shown how a power-law disk can support stable equilibrium families of eccentric orbits that align with the lopsidedness of the disk, as they reproduce its eccentricity profile. We further showed how, in such a disk, particles that librate in the AI are stable to off-plane perturbations, maintaining disk alignment. This is all encouraging, in the sense that it suggests that a fully self-consistent, thick, lopsided, and precessing disk can be constructed.

We would very much like to carry over our dynamical analysis to self-consistent equilibrium disks. For now, we note that, in such disks, a dispersion of apse directions will surely replace the apse-aligned eccentricity profiles of the present work. Apse dispersion, in the same razor-thin disks, will mainly contribute to enhance the potential contribution of the axisymmetric mode over the lopsided one. Such relative adjustments are expected to leave the present qualitative picture pretty much unchanged, all the while inducing variations in the eccentricities of the various equilibrium families. The extreme, of course, is a disk that is hot enough to have a uniform distribution in the apses, an axisymmetric disk that, depending on its radial density profile, will sustain a degenerate family of equilibria. Slight non-axisymmetry will then break the degeneracy and nucleate families of aligned and antialigned equilibria akin to the ones shown in Figures 3 and 11.

Comment on Odd TNOS. Currently, three of the eccentric, inclined TNOs with *a_p_* > 250 au *q_p_* > 30 au fall outside of ϖ_p_-confinement that we have sought to account for in terms of an antialigned, massive, eccentric trans-Neptunian disk. Here, we briefly review how these objects were analyzed with Planet Nine in the picture, as we further situate them within the phase space structured by DM1 and giant planets:

1. 2013 FT_28_ (Sheppard & Trujillo 2016) and 2015 KG_163_ (Shankman et al. 2017b): These two objects have apseorientations that are nearly antialigned with the clustered bunch of 10. For Planet Nine activists, the detection of these two objects was reassuring, for it was understood early on that an eccentric and inclined super-Earth would shelter stable eccentric objects with Δϖ = 0, i.e., that are planet-aligned in apse (Batygin & Brown 2016; Beust 2016). Sheppard & Trujillo (2016) take 2013 FT_28_ as symptomatic of a larger cluster (dubbed the “secondary cluster”) of TNOs that are stabilized in aligned orientations by/with Planet Nine. Batygin & Morbidelli (2017) pointed out that the alignment of FT_28_ and KG_163_ might be transient with a relatively short lifetime (100–500 Myr). We have here argued that DM1 shelters a family of stable aligned equilibrium orbits of moderate eccentricity that share the disk’s eccentricity profile. While discussing planar phase-space dynamics (Section 2), we have shown how this family of orbits seeds aligned islands of stability (the so-called AIs) in which particles undergo periodic oscillations in eccentricity and ϖ around the parent orbit. We further pointed out that members of the AI (and of the A-AI) find themselves on orbits that bring them close to the unstable aligned orbit, where they will tend to linger in transient disk-aligned states (see Figure 4). While it is tempting to suggest that 2013 FT_28_ and 2015 KG_163_ are in similar such transient states,^10^ lingering around the family of unstable aligned orbits, only further analysis with variants of DM1 (broad enough to include 2015 KG_163_) can determine that.
2. 2015 GT_50_ (Shankman et al. 2017b): A nonaligned TNO, almost orthogonal to the preferred apse orientation, which, for Shankman et al. (2017b), is yet another indication that ϖ confinement is due to observational bias (more on bias below). According to Batygin & Morbidelli (2017), 2015 GT_50_ is one in a class of eccentric objects that are predominantly controlled by Planet Nine on orbits with circulating longitude of the apse. We again refer to the discussion of planar phase-space dynamics in Section 2 to remind the reader that DM1, together with the outer planets, orchestrates a copious population of highly eccentric apse-circulating orbits, at semimajor axes that keep them safely out Neptune’s way. In particular, clones of 2015 GT_50_ within our model demonstrate circulatory behavior in their ϖ_*p*_ while undergoing small- amplitude oscillations in their orbital inclination and maintaining perihelion distance marginally larger than Neptune’s orbital radius.

**Comment on Observational Bias**. Shankman et al. (2017b) scrutinized observational bias in the OSSOS sample and concluded that there is no evidence of clustering in *ω*_p_ Ω_p_, and ϖ_p_ distributions. A similar conclusion was drawn by Lawler et al. (2017). Indeed, Shankman et al. (2017b) report that, although the OSSOS survey is biased toward detecting TNOs with ϖ_*p*_ near the region of observed clustering, it was able to detect TNOs across all values of ϖ, 2015 GT_50_ included (Shankman et al. 2017b).

On the other hand, Brown (2017) concluded that, although the observed sample is not free of biases, the statistical significance of the signal remains solid. Indeed, Brown (2017) estimates a rather low probability (∼1.2%) for the observed sample (with *a_p_* > 230 au) to be drawn from a uniform population.

Controversy over observational bias may or may not remove the need for a shepherding mechanism responsible for the spatial alignment noted by Batygin & Brown (2016). Additional TNO discoveries will surely help clarify the matter further. Here, we would like to build on the *e_p_*–a_p_ relationship noted and explored in our secular models (for instance, see Equation (15) and Figure 3) to propose the following: if it proves that further (seemingly) clustered TNOs maintain the *e_p_*–*a_p_* trend currently correlated with dynamical models, then we take that as a strong observational signature favoring secularly induced clustering in the presence of a massive disk, an outer planet, or both. Alternatively, if the *e_p_*–*a_p_* distribution of such objects reveals significant scatter, above and beyond that implied by the eccentricity-inclination dynamics of our models, then we would take that to weaken the case for dynamical clustering and weigh more in favor of bias in the observed clustering.

## 6 Conclusion

We have probed dynamical behavior stimulated by a relatively massive disk of icy bodies in trans-Neptunian space to flesh out a hunch concerning the interplay between the retrograde apse precession induced by such a disk and prograde precession forced by the outer planets: what if the clustered TNO population inhabits regions of phase space where the two effects cancel?

Analysis of coplanar dynamics yielded a family of eccentric, clustered, and apse frozen orbits, which showed remarkable agreement with the observed eccentricity-semimajor axis distribution. It further yielded a family of low-eccentricity orbits, aligned with the disk, which if properly populated, is expected to reproduce the disk that helps sustain them: a self- consistency argument that we require for our disk’s mass and eccentricity distributions.

We then allowed for out-of-plane motion, learned that an eccentric disk promotes linear stability to vertical motion, and gave evidence for the persistence of the planar backbone of stable apse-aligned orbits in inclined dynamics. We further analyzed the orbital evolution of the observed population of spatially aligned, eccentric, and inclined bodies, without addressing its origin, and concluded that the envisioned self- gravitating disk maintains what we like to think of as robust observables (i.e., eccentricity, inclination, longitude of apse) over the age of the solar system.

We carried out orbital simulations over the age of the solar system while assuming a fixed planetary configuration and a stationary disk. We ignored a dissipating gaseous disk, planetary migration and/or the scattering of objects into the region where our disk resides. We were naturally concerned with the range of behavior sustained in the “present” phase space of our hypothesized system. However, a massive gaseous disk could initially quench an eccentricity-inclination instabil- ity in a kinematically hot debris disk; then, with its dissipation, the instability kicks in and allows an initially axisymmetric disk to settle into a thick lopsided configuration that could harbor the apse-aligned orbits that we observe (M. Kazandjian et al. 2018, in preparation). Furthermore, migration of planets and secular resonances sweeping along with them might play a role in stirring an extended disk into an eccentric configuration (e.g Hahn & Malhotra 2005, and references therein).^11^

Our endeavor takes observational “evidence” for granted. Shankman et al. (2017a) cast doubt on the significance of the signal, further arguing that the mere observation of clustered TNOs, when taken at face value, requires a massive (~6-24 *M*_⊕_) extended reservoir of TNOs. We are, of course, happy to hear about indications for a massive population of TNOs, while our colleagues see it as problematic given the currently favored estimates for mass in this region of the solar system. These estimates put the total mass at 0.1 ~ *M*_⊕_ (Gladman et al. 2011). They are largely based on empirically constrained size distributions with significant uncertainty in their tails. We question those estimates and point to recent global simulations of protoplanetary disks suggesting the production of rather massive ($\underset{\sim }{≻}$ 60 *M*_⊕_) planetesimal disks beyond 100 au (Carrera et al. 2017).

The last thorough attempt at dynamical modeling of baryonic dark matter in the outer solar system was undertaken in the early 1990s (Hogg et al. 1991). By considering variations in cometary orbital elements, Hogg et al. (1991) argued for a few (perhaps hundreds of) Earth masses on scales of 100 au. We like to think that this early exercise (which incidentally was undertaken to carefully examine the evidence—or lack thereof —for a tenth planet) is being revisited piecemeal with Planet Nine in mind (Bailey et al. 2016; Batygin & Brown 2016; Fienga et al. 2016). We propose that similar such indirect measures be undertaken with an extended, moderately eccentric, disk of a few Earth masses perhaps replacing, perhaps combined with, a trans-Neptunian planet.

Of course, we can draw comfort in our hypothesis from observations of extra-solar debris disks, particularly massive ones around planet-hosting stars,^12^ as we hope for the mechanism of ϖ-confinement identified in this study to shed light on the dynamics and structure of these disks.

Ultimately, though, we do not have secure and direct observational evidence for our proposed disk, in much the same way we do not have full proof arguments against Planet Nine. Still, we hope to have given sufficiently many dynamical indicators for the game-changing role of such a disk in shepherding eccentric TNOs over a broad range of semimajor axes. Of course, a massive eccentric disk could operate simultaneously with a post-Neptunian planet to assure full secular spatial confinement if and when called for—and the converse is also true, in the sense that, with the proper mass distribution and orbital architecture, a disk-planet combination may prove capable of stabilizing orbits in configurations that are difficult to maintain with Planet Nine acting alone. TNO 2013 SY_99_ (Bannister et al. 2017) provides a case in point. A newly discovered object, its orbit is highly eccentric, apparently clustered with the wild bunch—but unlike its companions, nearly in the ecliptic with ∼4° orbital inclination. This object is so tenuously held on its orbit that it is exposed to the randomizing influence of Neptune, promoting diffusion at the inner edge of the Oort cloud. Interestingly enough, a Planet Nine-like influence is not of much stabilizing help here. In fact, when allowance is made for an inclined eccentric ninth planet, à la Batygin & Brown (2016), all hell breaks loose in the orbital evolution of this curious TNO (Bannister et al. 2017). Well, forgetting about Planet Nine for the moment, and entertaining, as we like to do, the possibility of a massive trans-Neptunian disk, we naturally find stable antialigned coplanar equilibria at a few hundreds astronomical unit, and with nearly the observed eccentricity! In fact, following the orbital evolution of 2013 SY_99_ under the action of our hypothesized disk, we learned that its current orbit can be sustained over the age of the solar system, executing only mild oscillations in inclination and perihelion distance, while maintaining near-alignment in ϖ as the pericenter and node circulate. These two limits, along with the eccentricity-semimajor axis distribution we have highlighted, speak in favor of the combined action of a self-gravitating trans-Neptunian disk, together with a trans-Neptunian terrestrial core (e.g., something akin to what was recently suggested by Volk & Malhotra (2017), or perhaps the result of a scattering event à la Silsbee & Tremaine (2018)). We conclude with the hope for this combined action to be the subject of parametric studies akin to the ones undertaken with Planet Nine acting alone.

This work grew out of a graduate seminar on the Oort Cloud conducted in Fall 2016 at the American University of Beirut. Discussions with participants M. Khaldieh and R. Badr are gratefully acknowledged. The authors would like to thank S. Tremaine, S. Sridhar, L. Klushin, and M. Bannister for helpful discussions, and an anonymous referee for constructive comments. We thank M. Kazandjian for making his modal analysis toolbox available to us. J.T. acknowledges insightful comments by R. Touma concerning the potential interplay between Planet Nine and a massive eccentric disk. A.S. acknowledges a scholarship by the Gates Cambridge Trust (OPP1144). Open Access for this article was funded by the Bill & Melinda Gates Foundation.

## Figures and Tables

**Figure 14:**
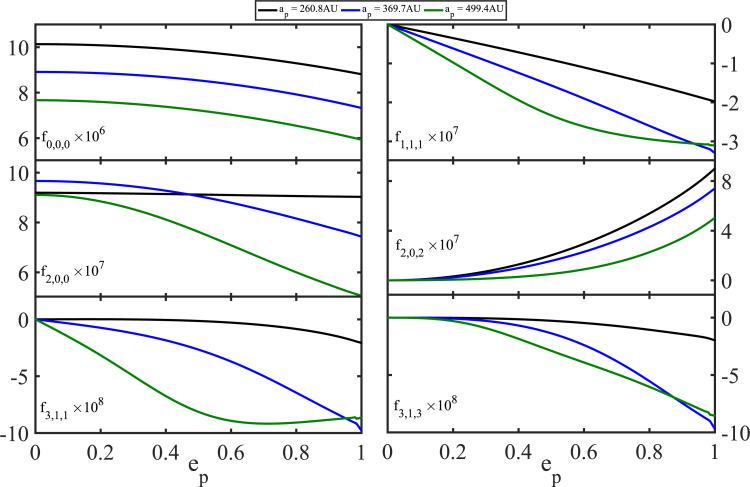
The behavior of numerically averaged functions *f_l,m,n_* = < *al,_m_* (*r*) cos(*nf*)> *a^p^*pearing in the disk potential (Equation (39)) as a function of eccentricity *e_p_* for orbits with different semimajor axes *a^p^*, as shown. The calculation is performed for the fiducial disk configuration DM1 (see Table 1). The chosen values of *a^p^* correspond to semimajor axes at which TNOs are observed (see Table 3). Note that the transition in behavior of fl,m,n with varying semimajor axis is smooth.

## Footnotes

3 The approximate expression of ϖ_p∣disk_ is obtained for circular disks with *p* = 1 under the reasonable assumption of *a_in_* ≪ *a_p_* ≪ *a_out_* . This assumption allows us to drop contributions to *R_d_* (Equation (4)) from the disk edges, rendering the coefficients ψ_i_ mild functions of only *p* and *q* (see Equations (31)–(36)). For instance, in a circular disk, ψ_2_ = −0.5 for p = 1; see Equation (32).

4 We know of two highly eccentric TNOs— 2013 FT_28_ ( (Sheppard & Trujillo 2016) and 2015 KG_163_ (Shankman et al. 2017b)—having *a_p_* < 250 au and *q_p_* < 30 au, which are currently antialigned with the much-discussed clustered bunch. Their dynamical behavior is reviewed in Section 5.

5 Similar such orbits, which tend to linger around eccentric aligned orientation, can be found in the A-AI when one moves sufficiently far from the equilibrium. These orbits are fairly eccentric, with some suffering encounters with Neptune on their journey.

6 The coplanar dynamics we just mapped out is naturally in dialog with Beust (2016), who considers a secular dynamics that is controlled by the outer planets together with a coplanar, eccentric, Planet Nine-like object. He identifies eccentric anti-aligned secular equilibria that are analogous to the ones we recover here. Perhaps the analogy can be pushed further to argue for the combined action of a pre-existing disk and a scattered planet. We discuss this possibility, but do not explore its detailed workings in the present article.

7 Performing the study at all positions in the disk, and not only at the seven considered semimajor axes (Table 3), does not affect the conclusions drawn from the population studies around AI and A-AI.

8 Important to note that Equation (15) captures the trend of growing equilibrium *e_p_* with a_p_, although the relation was derived for e_d_ = 0. Also note that increasing a_out_ (at constant M_d_ with *p* < 2 tends to lower equilibrium eccentricities.

9 This characteristic timescale can be reduced by at least an order of magnitude upon accounting for the disk and TNO eccentricities (see Equation (9)) for a given disk mass. Furthermore, more massive discs drive faster precession because *M_d_* ∝ (see Equation (10)).

10 Simulations of coplanar particles with the semimajor axis of 2013 FT_28_ (*a_p_* = 310.1 au) show lingering around the unstable aligned equilibrium (Δϖ = 0) for more than 5 Gyr when those particles are initiated in the AI or the A-AI (of Figure 4) on orbits with large enough amplitudes to bring them close to that unstable equilibrium. Naturally, such orbits maintain eccentricities akin to that of the unstable equilibrium (∼0.71; Figure 3), which is not so different from that of the inclined TNO 2013 FT_28_ (≈0.86).

11 To the perspicacious reader who wonders about the evolution of equilibrium families with migrating outer planets, we note that such migration will primarily modify the strength of planetary quadrupolar forcing, thus shifting the location of zero net apse precession in the disk and moderately perturbing the eccentricities of aligned and antialigned equilibria (∼10–20% in the course of migration).

## Appendix A

### The Disturbing Potential Due to the Disk

We present explicit expressions for the expansion of the orbit-averaged disturbing function (per unit mass) *R_d_* due to a disk composed of coplanar, apse-aligned, and confocal ellipses given in Equation (4). The expansion is carried out to fourth order in test-particle eccentricity, generalizing the work of Silsbee & Rafikov (2015), and is valid for arbitrary semimajor axes via the aid of the classical (unsoftened) Laplace coefficients. The mathematical details and techniques involved, such as expanding the disturbing function in eccentricities and retaining the secular part, are discussed in Sefilian (2017).

We consider a finite razor-thin disk, orbiting a central massive object, composed of confocal eccentric apse-aligned streamlines. The non-axisymmetric surface density of such a disk can be written as (Statler 1999),

(17) $$\Sigma \left(a_{d},\phi_{d}\right)=\Sigma_{d}\left(a_{d}\right)\times \frac{1-e_{d}{\left(a_{d}\right)}^{2}-a_{d}e_{d}^{\prime }\left[1+e_{d}\left(a_{d}\right)\right]}{1-e_{d}{\left(a_{d}\right)}^{2}-a_{d}e_{d}^{\prime }\left[e_{d}\left(a_{d}\right)+\cos E_{d}\right]}$$

where *E_d_* (ϕ_d_) is the eccentric (true) anomaly of the disk element and $e_{d}^{'}\equiv \frac{d}{da_{d}}e_{d}\left(a_{d}\right)$.

In what follows, we have assumed power-law relations for Σ*_d_*(*a_d_*) as given in Equations (1) and (2), although it is possible to use the same methods involved to recover expansions for arbitrary profiles as well as for cases with ϖ_*d*_ = ϖ_d_ (*a_d_*) (Ogilvie 2001; Statler 2001).

Our aim is to compute the secular potential Φ_*d*_ experienced by a test particle embedded in the disk,

(18) $$\Phi_{d}=-R_{d}=-G<\int \frac{\Sigma \left(r_{d},\phi_{d}\right)r_{d}dr_{d}d\phi_{d}}{\Delta }>$$

where the integration is performed over the area of the eccentric disk <…< represents time-averaging over the test-particle orbit, G is the gravitational constant, and $\Delta^{2}=r_{p}^{2}+r_{d}^{2}-2r_{p}r_{d}\cos\;\theta$, with *r*_p_ and *r_d_* being the instantaneous position vectors of the test particle and disk element, respectively, such that (*r^p^, r_d_*) = θ.

Following the classical formulation developed by Heppenheimer (1980), who first computed Ф_*d*_ for axisymmetric disks without softening the kernel Δ (see also, Ward 1981), we make use of the techniques laid down in Silsbee & Rafikov (2015) and extend their derivation to fourth order in eccentricities, rendering our formulae applicable in general astrophysical setups—which need not be cases where the assumption of *e* ≪ holds.

After a laborious series of algebraic manipulations (expand- ing, averaging, etc.), and ignoring constant terms that have no dynamical effect at the secular level, we find that Φ_*d*_ takes the following form:

(19) $$\Phi_{d}=-R_{d}=-K\left[\psi_{1}e_{p}\cos\left(\varpi_{p}-\varpi_{d}\right)+\left(\psi_{2}+\psi_{3}\cos\left(2\varpi_{p}-2\varpi_{d}\right)\right)e_{p}^{2}+\psi_{4}e_{p}^{3}\cos\left(\varpi_{p}-\varpi_{d}\right)+\psi_{5}e_{p}^{4}\right]$$

where (Equation 5a) and the dimensionless coefficients *ψ_i_* depend on α_1_ ≡ *a*_in_/*a*_out_, and the power-law indices p and q. To compactify the expressions of *ψ_i_*, we use the definition of Laplace coefficients

(20) $$b_{s}^{m}(\alpha )=\frac{2}{\pi }\int_{0}^{\pi }\;\frac{\cos(m\theta )}{{\left(1+\alpha^{2}-2\alpha \cos\theta \right)}^{s/2}}d\theta$$

and define the following auxiliary functions

(21) $$I(x,y,z)=\int_{x}^{1}\;\alpha^{y}b_{1}^{z}(\alpha )d\alpha \;\;\mathrm{and}\;\;D_{i}^{m}[f]=\frac{d^{m}}{d\alpha_{i}^{m}}f\left(\alpha_{i}\right)\;\;\mathrm{for}\;i=1,2\;\;\mathrm{and}\;\;m≽0$$

to write

(22) $$J_{1}=I\left(\alpha_{1},1-p,0\right)+I\left(\alpha_{2},p-2,0\right)$$

(23) $$J_{2}=I\left(\alpha_{1},1-p-q,0\right)+I\left(\alpha_{2},p+q-2,0\right)$$

(24) $$J_{3}=I\left(\alpha_{1},1-p-q,1\right)+I\left(\alpha_{2},p+q-2,1\right).$$

These definitions allow us to cast the coefficients following form:

(25) $$\psi_{1}=\frac{e_{d}\left(a_{p}\right)}{2}\left\{-(p+q)(p+q-3)J_{3}+\alpha_{2}^{p+q-1}\left[(2-p-q)D_{2}^{0}b_{1}^{1}+\alpha_{2}D_{2}^{1}b_{1}^{1}\right]\alpha_{1}^{2-p-q}\left[(p+q-1)D_{1}^{0}b_{1}^{1}+\alpha_{1}D_{1}^{1}b_{1}^{1}\right]\right\}+e_{d}{\left(a_{p}\right)}^{2}(p+2q)\left[\alpha_{2}^{p+2q-1}\left(D_{2}^{0}b_{1}^{1}+\frac{\alpha_{2}}{2}D_{2}^{1}b_{1}^{1}\right)-\frac{\alpha_{1}^{2-p-2q}}{2}\left(D_{1}^{0}b_{1}^{1}-\alpha_{1}D_{1}^{1}b_{1}^{1}\right)\right]$$

(26) $$\psi_{2}=\frac{\left(1-p)(2-p\right)}{4}J_{1}+\frac{1-p}{2}\alpha_{1}D_{1}^{0}\left[\alpha_{1}^{1-p}b_{1}^{0}\right]-\frac{\alpha_{1}^{2}}{4}D_{1}^{1}\left[\alpha_{1}^{1-p}b_{1}^{0}\right]+\frac{p-2}{2}\alpha_{2}D_{2}^{0}\left[\alpha^{p-2}b_{1}^{0}\right]-\frac{\alpha_{1}^{2}}{4}D_{2}^{1}\left[\alpha^{p-2}b_{1}^{0}\right]+\frac{e_{d}\left(a_{p}\right)}{4}\left[q(1-p-q)(2-p-q)J_{2}+2q(1-p-q)\times \alpha_{1}D_{1}^{0}\left[\alpha^{1-p-q}b_{1}^{0}\right]-q\alpha_{1}^{2}D_{1}^{1}\left[\alpha^{1-p-q}b_{1}^{0}\right]+2q(p+q-2)\alpha_{2}D_{2}^{0}\left[\alpha^{p+q-2}b_{1}^{0}\right]-q\alpha_{2}^{2}D_{2}^{1}\left[\alpha^{p+q-2}b_{1}^{0}\right]+2\alpha_{2}^{p+q}\;\left(D_{2}^{1}b_{1}^{0}+\frac{\alpha_{2}}{2}D_{2}^{2}b_{1}^{0}\right)-2\alpha_{1}^{3-p-q}\left(D_{1}^{1}b_{1}^{0}+\frac{\alpha_{1}}{2}D_{1}^{2}b_{1}^{0}\right)\right]+e_{d}{\left(a_{p}\right)}^{2}\frac{p+3q}{4}\left[\alpha_{2}^{p+2q}\left(D_{2}^{1}b_{1}^{0}+\frac{\alpha_{2}}{2}D_{2}^{2}b_{1}^{0}\right)-\alpha_{1}^{3-p-2q}\left(D_{1}^{1}b_{1}^{0}+\frac{\alpha_{1}}{2}D_{1}^{2}b_{1}^{0}\right)\right]$$

(27) $$\psi_{3}=e_{d}{\left(a_{p}\right)}^{2}\frac{p+q}{8}\left[3\alpha_{2}^{p+2q-1}\left(D_{2}^{0}b_{1}^{2}+\alpha_{2}D_{2}^{1}b_{1}^{2}+\frac{\alpha_{2}^{2}}{6}D_{2}^{2}b_{1}^{2}\right)-\alpha_{1}^{2-p-2q}\left(D_{1}^{0}b_{1}^{2}-\alpha_{1}D_{1}^{1}b_{1}^{2}+\frac{\alpha_{1}^{2}}{2}D_{1}^{2}b_{1}^{2}\right)\right]$$

(28) $$\psi_{4}=\frac{e_{d}\left(a_{p}\right)}{16}\left[(p+q{)}^{2}(1-p-q)(p+q-3)J_{3}-(p+q)[(p+q-2)(3p+3q-5)-2]\times \alpha_{2}D_{2}^{0}\left[\alpha^{p+q-2}b_{1}^{1}\right]+(p+q)(3p+3q-7)\times \alpha_{2}^{2}D_{2}^{1}\left[\alpha^{p+q-2}b_{1}^{1}\right]-(p+q)\alpha_{2}^{3}D_{2}^{2}\left[\alpha^{p+q-2}b_{1}^{1}\right]+(p+q{)}^{2}(3p+3q-5)\alpha_{1}D_{1}^{0}\left[\alpha^{1-p-q}b_{1}^{1}\right]+(p+q)(3p+3q-1)\alpha_{1}^{2}D_{1}^{1}\left[\alpha^{1-p-q}b_{1}^{1}\right]+(p+q)\alpha_{1}^{3}D_{1}^{2}\left[\alpha^{1-p-q}b_{1}^{1}\right]+\alpha_{2}^{p+q+1}\left(4D_{2}^{2}b_{1}^{1}+\alpha_{2}D_{2}^{3}b_{1}^{1}\right)-2\alpha_{1}^{2-p-q}\left(D_{1}^{0}b_{1}^{1}-\alpha_{1}D_{1}^{1}b_{1}^{1}\right.\left.-\frac{5\alpha_{1}^{2}}{2}D_{1}^{2}b_{1}^{1}-\frac{\alpha_{1}^{3}}{2}D_{1}^{3}b_{1}^{1}\right)\right]$$

and

(29) $$\psi_{5}=\frac{p\left(1-p^{2}\right)(2-p)}{64}J_{1}-\frac{\alpha_{1}}{16}p\left(p^{2}-1\right)D_{1}^{0}\left[\alpha^{1-p}b_{1}^{0}\right]-\frac{3\alpha_{1}^{2}}{32}\left(p^{2}+p\right)D_{1}^{1}\left[\alpha^{1-p}b_{1}^{0}\right]-\frac{\alpha_{1}^{3}}{16}(p+1)\times D_{1}^{2}\left[\alpha^{1-p}b_{1}^{0}\right]-\frac{\alpha_{1}^{4}}{64}D_{1}^{3}\left[\alpha^{1-p}b_{1}^{0}\right]+\frac{\alpha_{2}}{16}\left(p^{3}-3p^{2}+2p\right)D_{2}^{0}\left[\alpha^{p-2}b_{1}^{0}\right]-\frac{3\alpha_{2}^{2}}{32}(p-1)(p-2)D_{2}^{1}\left[\alpha^{p-2}b_{1}^{0}\right]-\frac{\alpha_{2}^{3}}{16}(2-p)\times D_{2}^{2}\left[\alpha^{p-2}b_{1}^{0}\right]-\frac{\alpha_{2}^{4}}{64}D_{2}^{3}\left[\alpha^{p-2}b_{1}^{0}\right].$$

We note that the disk potential Φd presented here reduces to that of Silsbee & Rafikov (2015) upon limiting it to second order in eccentricities, and naturally to that of Heppenheimer (1980) for axisymmetric disks.

We end with a brief remark about the behavior of the coefficients Φi. The expressions of Φi depend on the disk boundaries through α1 and α2 such that the magnitudes of Φi diverge when the test-particle is situated nearby the disk edges. However, in the opposite limit, when the test-particle semimajor axis is well-separated from the disk boundaries *a*_in_ and *a*_out_, we can ignore the edge effects, provided that the disk spans several order of magnitude in radius. In such a case (α_1_, α_2_ $\underset{\sim }{≻}$ 0), we can get relatively simple closed-form expressions for *Φ_i_* that are valid for *a_in_* < < *a_p_* < < *a*_out_ by using the series expansions of complete elliptic integrals (Gradshteyn & Ryzhik 1994), such that:

(30) $$\psi_{1}=e_{d}\left(a_{p}\right)\left[\frac{3}{2}-(p+q)(p+q-3)\times \sum_{n=2}^{\infty }\;\frac{2nA_{n}(4n-1)}{\left(2n-1)(2n+1-p-q)(2n+p+q-2\right)}\right]$$

(31) $$\psi_{2}=-\frac{1}{2}+\frac{\left(1-p)(2-p\right)}{2}\times \sum_{n=1}^{\infty }\;\frac{(4n+1)A_{n}}{\left(2n+2-p)(2n+p-1\right)}$$

(32) $$+\frac{qe_{d}\left(a_{p}\right)}{2}\left[1+(1-p-q)(2-p-q)\times \sum_{n=1}^{\infty }\;\frac{(4n+1)A_{n}}{\left(2n+2-p-q)(2n+p+q-1\right)}\right]$$

(33) $$\psi_{3}=0$$

(34) $$\psi_{4}=\frac{\left(p+q)(p+q-1\right)}{8}\psi_{1}$$

(35) $$\psi_{5}=\frac{p(p+1)}{16}\psi_{2}l_{q=0}$$

## Appendix B Numerical 3D Potential of a Disk

Here, we present a brief recipe to numerically recover the full 3D gravitational potential generated by a disk formed of coplanar, apse-aligned, eccentric rings. The numerical tool that we developed is indeed general and can be employed to recover the potential due to any configuration of rings (warped, inclined, spiral-armed disks, etc.). The ramifications of this tool will be explored in the future (M. Kazandjian et al. 2018, in preparation).

For the purposes of this study, we distributed *N* (=1024) coplanar, apse-aligned rings over the range of the disk with specified mass and eccentricity distributions (Equations (1) and (2)). We then computed the full 3D potential experienced by test particles by numerically recovering the associated spherical harmonics *Y*_l_^m^(*θ, ϕ*). Identifying the most dominant modes *a_1,m_* (*r*), we then numerically orbit-averaged the arising terms and obtained closed-form expressions for the modes in question for any given semimajor axis.

This numerical procedure allows us to express the secular potential of any disk as

(36) Φd=∑l,m <al,m(r)Ylm(θ,ϕ)>

where <…> stands for time-averaging. The angles θ and ϕ can be expressed in terms of the usual orbital elements by using

(36a) $$x=r\;\sin\theta \;\cos\phi =r\left[\cos\left(\Omega -\varpi_{d}\right)\cos(\omega +f)-\sin\left(\Omega -\varpi_{d}\right)\sin(\omega +f)\cos(i)\right]$$

(36b) $$y=r\;\sin\theta \;\sin\phi =r\left[\sin\left(\Omega -\varpi_{d}\right)\cos(\omega +f)+\cos\left(\Omega -\varpi_{d}\right)\sin(\omega +f)\cos(i)\right]$$

(36c) z=r cosθ=r sin(ω+f)sin(i)

where *f* is the true anomaly.

Equipped with the relevant dominant modes *a_l,m_*(*r*), we find that the potential due to the reference disk (DM1; Table 1) takes the following form:

(37) Φd=−14π<a0,0(r)>+54π<a2,0P20(cosθ)>−38π<a1,1(r)P11(cosθ)cosϕ>+748π<a3,1(r)P31(cosθ)cosϕ>

where we have assumed that we have averaged over the orbit of the test particles (<̤>) and *P_l_^m^*(*x*) are the Legendre polynomials.

After long and tedious but straightforward algebra, we express Φ_*d*_ in terms of the orbital elements and write

(38) Φd=Fep+GepcosΩp−ϖdcosωp−cosipsinΩp−ϖdsinωp+3454πsin2ipf2,0,0−cos2ωpf2,0,2+3548cosΩp−ϖdcos3ωpf3,1,3−cosωpf3,1,1+158748πcosipsin2ipsinΩp−ϖd×sinωp3f3,1,1−f3,1,1−2f3,1,3cos2ωp

where *f_l,m,n_*(*e_p_*; *a_p_*) = á*a_l, m_*(*r*)cos(*nf*)ñ are computed numeri- cally at a given semimajor axis to the desired (arbitrary) order in eccentricity, and we have defined

(39a) $$F=-\sqrt{\frac{1}{4\pi }}f_{0,0,0}-\frac{1}{2}\sqrt{\frac{5}{4\pi }}f_{2,0,0}$$

(39b) $$G=\sqrt{\frac{3}{8\pi }}f_{1,1,1}+\frac{3}{2}\sqrt{\frac{7}{48\pi }}f_{3,1,1}$$

It is noteworthy that all coplanar, apse-aligned, power-law disks share this general form of the Hamiltonian, with the only difference being in the functions *a_l,m_*(*r*) that depend on the characteristics of the disk: mass distribution, eccentricity profile, and the boundaries. For reference, in Figure 14 we show the time-averaged behavior of the functions *f_l,m,n_* for DM1 as a function of test-particle eccentricity at three values of semimajor axes.

With the orbit-averaged mean field of the razor-thin eccentric disk in hand, we add the secular contribution of the outer planets and write the total Hamiltonian H as

(40) H=Φd−Γ6lp33cosip2−1

where Γ is given by Equation (7).

This allows us to express the equations of motion governing the dynamics of a TNO under the effect of the considered disk and the giant planets as:

(41) $$L_{p}{\dot{\Omega }}_{p}=-C_{i}l_{p}^{-1}\left(\frac{\Gamma }{l_{p}^{3}}+\frac{3}{4}\sqrt{\frac{5}{\pi }}T_{1}+\frac{5}{16}\sqrt{\frac{21}{\pi }}C_{h}T_{2}\right)-S_{h}S_{\omega }l_{p}^{-1}\left[\frac{15}{32}\sqrt{\frac{21}{\pi }}C_{i}^{2}T_{3}+\left(G-\frac{5}{32}\sqrt{\frac{21}{\pi }}T_{3}\right)\right]$$

(42) $$L_{p}{\dot{l}}_{p}=G\left(C_{h}S_{\omega }+C_{i}S_{h}C_{\omega }\right)-\frac{5}{32}\sqrt{\frac{21}{\pi }}C_{i}S_{i}^{2}S_{h}\left(4S_{\omega }S_{2\omega }f_{3,1,3}+C_{\omega }T_{3}\right)-\frac{3}{4}S_{i}^{2}\left(\sqrt{\frac{5}{\pi }}S_{2\omega }f_{2,0,2}-\frac{5}{8}\sqrt{\frac{21}{\pi }}C_{h}S_{3\omega }f_{3,1,3}+\frac{5}{24}\sqrt{\frac{21}{\pi }}C_{h}S_{\omega }f_{3,1,1}\right)$$

(43) $$L_{p}{\dot{\alpha }}_{p}=G\left(S_{h}C_{\omega }+C_{i}C_{h}S_{\omega }\right)+\frac{5}{32}\sqrt{\frac{21}{\pi }}S_{i}^{2}\left(S_{h}T_{2}-C_{i}C_{h}S_{\omega }T_{3}\right)$$

(44) $$L_{p}{\dot{\omega }}_{p}=\frac{\Gamma }{6l_{p}^{4}}\left(15C_{i}^{2}-3\right)+Gl_{p}^{-1}C_{i}S_{h}S_{\omega }+\frac{5}{32}\sqrt{\frac{21}{\pi }}l_{p}^{-1}C_{i}\left(3C_{i}^{2}-1\right)S_{h}S_{\omega }T_{3}+l_{p}^{-1}C_{i}^{2}\left[\frac{3}{4}\sqrt{\frac{5}{\pi }}T_{1}+\frac{5}{16}\sqrt{\frac{21}{\pi }}C_{h}T_{2}\right]-\frac{l_{p}}{\sqrt{1-l_{p}^{2}}}\left[F^{\prime }+G^{\prime }\left(C_{h}C_{\omega }-C_{i}S_{h}S_{\omega }\right)+S_{i}^{2}\left(\frac{3}{8}\sqrt{\frac{5}{\pi }}T_{1}^{\prime }+\frac{5}{32}\sqrt{\frac{21}{\pi }}C_{h}T_{2}^{\prime }\right)+\frac{5}{32}\sqrt{\frac{21}{\pi }}S_{h}S_{\omega }C_{i}S_{i}^{2}T_{3}^{\prime }\right]$$

where we have written

(45a) $$L_{p}=\sqrt{GM_{⊙}a_{p}}$$

(45b) $$T_{1}=f_{2,0,0}-C_{2\omega }f_{2,0,2}$$

(45c) $$T_{2}=C_{3\omega }f_{3,1,3}-C_{\omega }f_{3,1,1}$$

(45d) $$T_{3}=3f_{3,1,1}-f_{3,1,3}\left(1+2C_{2\omega }\right)$$

Finally, we comment on the accuracy of our numerical disk potential. Insisting on coplanar test-particle orbits by setting *i_p_* = 0 in Equation (41), we solved for the equilibria and analyzed their stability. The results are shown in Figure 15. It is evident that the coplanar equilibria recovered by the numerical formulation of the disk potential (Equation (39)) and that of the analytical (Equation (4)) agree very well both in orbital structure and stability.
